# Assessment of Glucagon-Like Peptide-1 Analogue and Renin Inhibitor on the Binding and Regulation of GLP-1 Receptor in Type 1 Diabetic Rat Hearts

**DOI:** 10.1155/2011/489708

**Published:** 2011-06-04

**Authors:** Shushan B. Artinian, Sawsan M. Al Lafi, Suzan S. Boutary, Khalil M. Bitar, Nadine S. Zwainy, Anwar B. Bikhazi

**Affiliations:** ^1^Department of Physiology, Faculty of Medicine, American University of Beirut, Beirut 11-236, Lebanon; ^2^Department of Physics, Faculty of Arts and Sciences, American University of Beirut, Beirut 11-236, Lebanon

## Abstract

This study focuses on the effects of long-term renin-angiotensin system suppression and/or incretin mimetic therapies on the regulation and binding affinity of GLP-1 to its receptor in the coronary endothelium (CE) and cardiomyocytes (CMs) of type 1 diabetic male Sprague-Dawley rats. The groups assessed are normal (N), streptozotocin-induced diabetic (D), Insulin treated (DI), Exendin-4 treated (DE), Aliskiren treated (DA), cotreated with Insulin and Aliskiren (DIA) and cotreated with exendin-4 and Aliskiren (DEA). Heart perfusion with ^125^I-GLP-1 was performed to estimate GLP-1 binding affinity (*τ* = 1/k-n) to its receptor in the heart. Western Blotting was assessed to determine the expression variation of GLP-1 receptor in the heart. Plasma GLP-1 levels were measured using Enzyme-Linked Immunosorbent Assay (ELISA). Diabetes decreased the *τ* value on CE and increased it on CMs compared to normal. The combination of Exendin-4 with Aliskiren showed a normalizing effect on the binding affinity of GLP-1 at the coronary endothelium, while at the cardiomyocyte level Exendin-4 treatment alone was the most effective.

## 1. Introduction

Diabetes mellitus currently affects more than 170 million individuals worldwide [1]. Other than hyperglycemia, diabetes mellitus can cause a 2-3-fold increase in the occurrence of cardiovascular disease (CVD) [2]. Both manifestations are easily triggered by oxidative stress, glucose intolerance, and inflammation; hence, they probably exhibit similar underlying processes that lead to their pathogenesis [1]. The incretin hormone, glucagon-like peptide-1 (GLP-1), plays an important role in maintaining glucose homeostasis. Receptor signaling on the pancreas leads to enhanced insulin biosynthesis, secretion, and *β*-cell proliferation [3, 4]. GLP-1 has also been suggested to ameliorate left ventricular function, because of its antiapoptotic and insulin-like properties [5]. The incretin effect, described as the enhanced response of insulin release after an oral glucose load, has been shown to be reduced in diabetes mellitus [6]. This defect in GLP-1 secretion has been reported in both Type 1 and Type 2 diabetes mellitus [7]. On the other hand, a recent study reported that GLP-1 levels are not decreased in type 2 diabetic patients [8]. Therefore, the effect of diabetes mellitus on the secretion of GLP-1 is a controversial issue. The renin angiotensin system (RAS) controls and regulates the electrolyte-fluid homeostasis and blood pressure by acting on organs in the cardiovascular, renal, and adrenal systems [9]. Clinically, RAS blockage has been proposed to alleviate diabetic complications. In 2007, Aliskiren, a potent renin inhibitor, was approved for clinical use. It is unique due to its low molecular weight, its orally active property, and its nonpeptide nature that makes it resistant to enzymatic degradation [10, 11]. Aliskiren binds to renin, hence blocking the attachment of angiotensinogen to it and its consequent conversion to angiotensin I [12]. It was also shown to reduce left ventricular hypertrophy as efficiently as angiotensin receptor blockers (ARBs) [13]. This study aims to assess the effect of the GLP-1 analogue, Exendin-4, and the renin inhibitor, Aliskiren, and their cotreatment on the binding kinetics of GLP-1 to its receptor at both the coronary endothelial and cardiomyocyte levels in type 1 diabetic rats. 

## 2. Materials and Methods

The experiments were conducted with prior approval of the Institutional Review Board and Animal Care Committee of the American University of Beirut (AUB). All animals were handled, treated, and sacrificed in accordance with the guidelines of the American Association for Laboratory Animal Sciences (AALAS) on *Humane Care and Use of Laboratory Animals*. For all the parameters, per animal per group, mentioned below, the experimental data are presented as mean value ± standard error of mean (SEM). Student's *t*-test was employed to estimate the significance among the different experimental groups. *P* values of less than  .05 were considered significant. 

### 2.1. Animals

Male Sprague-Dawley rats (6 weeks old, 175–250 g body weight) were purchased from Harland, The Netherlands, and bred at the Animal House Unit, American University of Beirut. They were housed at four rats per cage (24 animals per group), fed Purina pellets and tap water ad libitum, and kept for a period of one month at a constant temperature with a daily 12 h light : 12 h dark cycle.

### 2.2. Treatment and Monitor Plan

Rats were divided into seven groups as follows: Group N (*n* = 24): normal control, received a placebo by oral gavage (tap water, 4 mL/kg body weight), once daily (qd); Group D (*n* = 24): rats with diabetes type 1 were injected intraperitoneally (ip) with 3 cc/kg body weight normal saline solution (NSS), twice daily (bid), and were given placebo (water) by oral gavage (4 cc/kg body weight, qd); Group DI (*n* = 24): rats with diabetes type 1 were injected ip with bovine insulin (Sigma Chemical Company, St. Louis, MI, USA), 0.28 unit/cc, 1 unit/kg body weight, once in the morning (qAM), and subcutaneous insulin glargine (Lantus) injections (1.25 unit/cc, 1 unit/kg body weight) (Sanofi-Aventis, USA), once in the afternoon (qPM); Group DE (*n* = 24): rats with diabetes type 1 were injected intraperitoneally (ip) with Exendin-4 (0.03 *μ*g/kg body weight, bid) (Sigma Chemical Company, St. Louis, Mich, USA); Group DA (*n* = 24): rats with diabetes type 1 were administered Aliskiren (50 mg/kg body weight, qd) (Novartis Pharma Stein AG, Switzerland) by oral gavage. Group DIA (*n* = 24): rats with diabetes type 1 were injected ip with bovine insulin qAM, injected subcutaneously insulin glargine qPM, and were administered Aliskiren (50 mg/kg body weight, qd) by oral gavage. Group DEA (*n* = 24): rats with diabetes type 1 were injected intraperitoneally (ip) with Exendin-4 and were administered Aliskiren (50 mg/kg body weight, qd) by oral gavage.

### 2.3. Induction of Diabetes

Groups D, DI, DE, DA, DIA and DEA were induced to type 1 diabetes mellitus by a single intravenous injection of streptozotocin (STZ; 85 mg/kg bw) (Sigma Chemical Co., Saint Louis, Mo, USA) in saline acidified to pH 4.5 with 0.1 M citrate buffer [14]. Three days later, nonfasting blood glucose level was measured using Accu-Chek (Accu-Chek Instant Test; Roche Diagnostics GmbH, Mannheim, Germany); a level of ≥250 mg/dL confirmed type 1 diabetes mellitus.

### 2.4. Body Weight and Blood Glucose

All the animals were weighed weekly, and blood glucose levels were determined [15] using Accu-Chek (Accu-Chek instant test, Roche Diagnostics GmbH, Mannheim, Germany) weekly during one month of treatment.

### 2.5. Cardiac Hypertrophy Was Assessed Macroscopically

After one month of treatment, wet heart weight was recorded (N = 16). Heart weight (H.W.) to body weight (B.W.) ratio (H.W./B.W.) was determined and averaged that served as an index for comparison among different groups.

### 2.6. Enzyme-Linked Immunosorbent Assay of GLP-1

**Figure 1:**
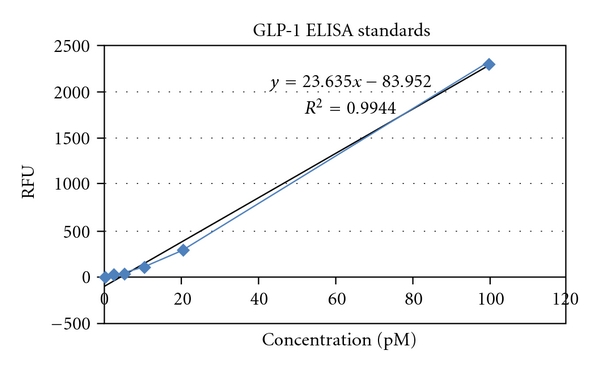
Standard curve for GLP-1.

Rats were anesthetized, and blood was collected from the sublingual vein on days 1, 7, 14, 21, and 28 of the treatment period, at a constant time range of 9 to 10 AM. For each 1 mL blood, 10 *μ*L of DPP-IV enzyme inhibitor was added within 30 seconds of the collection of blood to prevent GLP-1 degradation. Enzyme-Linked Immunosorbent Assay (ELISA) was performed to detect the levels of active GLP-1 (7–36 and 7–37) in the plasma of the rats using the Glucagon-Like Peptide-1 (Active) ELISA kit (ELISA KIT LINCO Research, Mish, USA). Standards of known concentrations of active GLP-1 and the samples were added to the 96-well plate. GLP-1 detection conjugate and substrate were added according to the kit manual to generate a reaction, and the plate was read on the Fluorescent Plate reader at wavelengths of 355/460 nm. A relative fluorescent unit (RFU) curve was plotted using these standards (Figure 1). Since the amount of fluorescence generated is directly proportional to the concentration of GLP-1, the concentration of GLP-1 in the plasma samples can be derived.

### 2.7. Surgical Procedures

After one month of treatment, the rats were weighed and anesthetized by intramuscular injection of Ketamine (100 mg/kg body weight) (AUB-MC, Lebanon) and Xylazine (10 mg/kg body weight) (Interchemie, Casternary, Holland) and then fixed to a heating pad to prevent rapid cooling. The anterior chest wall was excised longitudinally up to the xyphoid, thus exposing the entire thoracic cavity and the heart for perfusion [16]. 

### 2.8. Perfusion of Rat Heart with ^125^I-GLP-1

**Figure 2:**
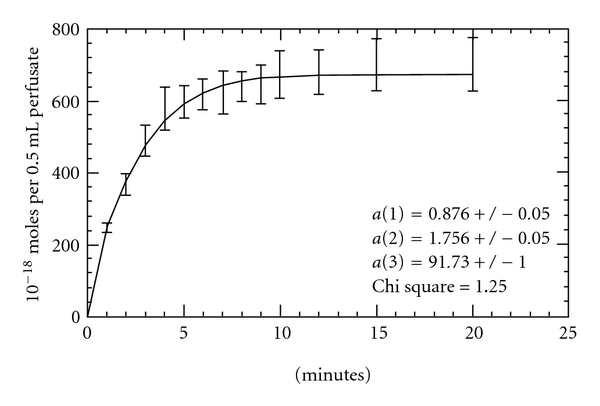
A representative time dependent [^125^I]-GLP-1 concentration curve in the effluent collected during heart perfusion in Normal group (N) at the level of endothelium. Data points were curve fitted using equation 2 as described by Haddad et al. [17]. The values *a*(1), *a*(2), and *a*(3) were employed to calculate *k*_*n*_ and *k*_−*n*_.

The major veins and arteries including the inferior, superior vena cava, pulmonary artery, and pulmonary vein were ligated. In addition, the left and right lungs were tied in order to block the blood flow to them and to other organs. The aorta was slightly cut, and a polyethylene catheter was inserted (from cephalad to caudad) into its lumen to reach the aortic valve. The right atrium was punctured, and another catheter was inserted to carry out the perfusate. Both catheters were secured with a suture [17]. A 50 mL syringe filled with heparinized Ringer-Lock buffer containing 20 meq/L K ^+^, oxygenated with 95% O_2_, 5% CO_2_ at 37°C linked to an infusion pump was attached to the inlet. This solution cleared the heart from blood and clots for 15 minutes; infusion of a Ringer-Lock buffer solution for another 15 minutes cleared the heart from heparin. These steps were followed by the perfusion of a buffer containing 8.25 × 10^−4^ nM/L [^125^I] GLP-1 (specific activity, 2200 Ci/mmol; Santa Cruz Biotech., Calif, USA) at a rate of 1 mL/min. Heart perfusion was performed in 16 rats from each set divided into one subgroup (*n* = 8) perfused with buffer alone; and the other (*n* = 8) perfused with 20 mmol/L 3-[(3-cholamidopropyl) dimethylammonio]-1-propanesulfonate (CHAPS; Sigma), which has a mild detergent action, to slough off the capillary endothelial lining. The perfusate was collected for 20 minutes at specific time intervals (between 0-1, 1-2, 2-3, 3-4, 4-5, 5-6, 6-7, 7-8, 8-9, 9-10, 10–12, 12–15, 15–20 minutes); after each collection, 500 *μ*L of the sample was put in a liquid scintillation vial containing 4 mL of Ecolume scintillation cocktail, which was later assayed for radioactivity by a liquid scintillation analyzer. The surgical procedure and perfusion model as described by Bikhazi et al. [16] and Haddad et al. [17] were followed to determine the binding kinetics of ^125^I-GLP-1 (specific activity, 2200 Ci/mmol; Santa Cruz Biotech., Calif, USA) to its receptor at the level of endothelial cells and cardiac myocytes (Figure 2).

### 2.9. Western Blot

After the thoracic cage was excised and the inferior vena cava was cut, a fine needle was inserted in the beating heart washing it several times with saline water. The heart was then removed and immediately put in a beaker containing isopentane and dry ice. When the heart was snap frozen, it was cut transversely into 4 sections: apex, S1, S2, and base. The S1 heart sections were later homogenized, and proteins were extracted using Sucrose Hepes Tris-Buffer and a serine protease inhibitor, PMSF. 150 *μ*g of the sample protein was loaded and separated by 10% polyacrylamide gel electrophoresis for 1 hour and 30 minutes. The bands were then transferred on a nitrocellulose membrane via the Transblot unit, PowerPac HC, (Bio-Rad Laboratories, Calif, USA) for 2 hours. The membrane was incubated with primary polyclonal antibody rabbit anti-GLP-1 receptor *α* IgG (Santa Cruz Biotech., Calif, USA) (diluted 1/200) for 1 hour. Three consecutive washes with Tris/Tween solution, after which the membrane was incubated with the secondary antibody, mouse antirabbit IgG-HRP 2° (Santa Cruz Biotech.) (1/500) for 2 hours, then washed 4 times with the same Tris/Tween solution. Equal volumes of reagents A and B of chemiluminescence solution were mixed and poured onto the membrane. The immunoblotted bands were later developed on a Fuji Medical X-ray film (Agfa-Gevaert N.V., Mortsel, Belgium). 

## 3. Results

### 3.1. Body Weights

**Figure 3:**
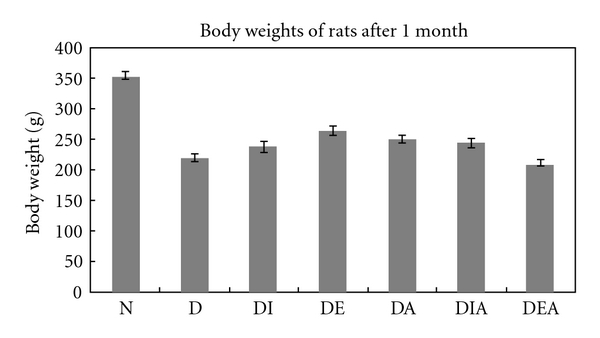
Body weights of rats in the seven experimental groups after 1 month of treatment (N = 24).

The mean body weights for the seven rat groups after one month of treatment are shown in Figure 3. One can observe the significant decrease (*P* < .001) in the body weight of all diabetic groups compared to the normal. 

### 3.2. Cardiac Hypertrophy Was Assessed Macroscopically

**Figure 4:**
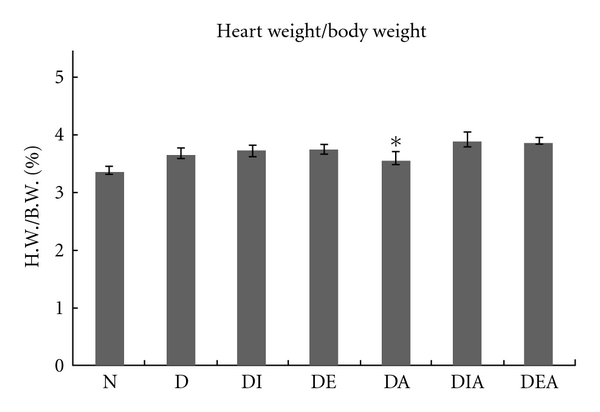
Mean ratios of heart weight per body weight of the seven rat groups after 1 month. *indicates no significance compared with normal group (N = 16).

The mean ratios of heart weight to body weights are represented in Figure 4. The data show a significantly increased heart weight to body weight ratio in all the diabetic groups compared to the normal (D, DI, DE, DIA, DEA) except the group treated with Aliskiren (DA).

### 3.3. Plasma Glucose and GLP-1 Levels

**Figure 5:**
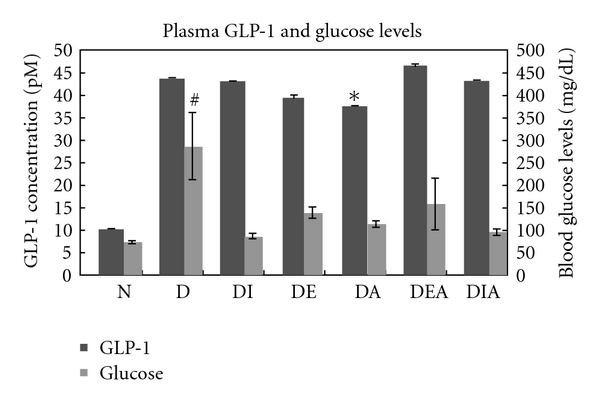
Plasma glucose (N = 24) and glucagon-like peptide-1 levels (N = 6) in all the animal groups after one month of treatment. *indicates significance with *P* < .05 compared with Glucose level of diabetic (D); ^#^indicates extreme significance with *P* < .001 compared with GLP-1 level of normal (N).

There was a significant increase in plasma glucose levels in all the diabetic groups compared to the normal. It is notable that 12.5% of the Aliskiren-treated group exhibited normal blood glucose levels after one month of treatment. GLP-1 levels in the plasma of normal rats were assessed by ELISA, and a range of 7–9 pM was observed with no significant variation throughout a month. Upon the induction of diabetes mellitus, however, GLP-1 levels increased with a maximum peak of 28 pM at day 28, compared to that of the normal. GLP-1 levels of diabetic rats treated with Insulin (DI), Exendin (DE), or Aliskiren (DA) were normalized. The combination of Aliskiren with Insulin (DIA) was shown to be better than the combination of Exendin-4 and Aliskiren (DEA), because it corrected and nearly normalized GLP-1 levels in the diabetic rats (Figure 5).

### 3.4. Binding Kinetics of GLP-1 to Its Receptor on the Coronary Endothelium and Cardiomyocytes after One Month of Treatment

Time-dependent radioactive GLP-1 ([^125^I]-GLP-1) concentration curves of all the animal models were mathematically curve-fitted using a first-order Bessel function physical model describing a 1 : 1 stoichiometry for reversible binding of GLP-1 with its receptor [16]. These curves were used to determine the GLP-1 forward binding constant (*k*_*n*_), reversal constant (*k*_−*n*_), dissociation constant (*K*_*d*_ = *k*_−*n*_/*k*_*n*_), and the affinity time constant (*τ* = 1/*k*_−*n*_) with its receptor on the coronary endothelium cells and cardiomyocytes [16, 17]. The derived *K*_*d*_ and *τ* values at the coronary endothelium are represented in Table 1, and those values at the cardiomyocyte level are represented in Table 2. One can notice the extremely significant increase in *K*_*d*_ constant in the diabetic untreated (D), treated with Exendin-4 (DE), treated with Aliskiren (DA), cotreated with Exendin-4 and Aliskiren (DEA), and cotreated with Insulin and Aliskiren (DIA) compared to the normal. However, diabetic treated with Insulin (DI) showed no significance compared to the normal. Diabetes decreased the *τ* value on CE and increased it on CM compared to normal. Exendin-4 treatment partially corrected *τ* value in both CE and CM. Aliskiren treatment did not alter *τ* from diabetics in the CE, but its combination with Exendin-4 normalized it.

### 3.5. Western Blot Analysis

**Figure 6:**
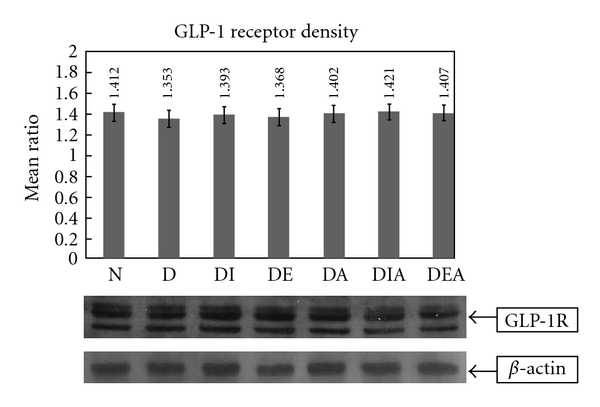
A representative Western blot GLP-1 receptor bands, *β*-actin bands and their mean ratio (N = 6).

Western blotting was assessed to determine the expression variation of GLP-1 receptor in the heart. The GLP-1 receptor band densities were measured using ImageJ program. The housekeeping gene, *β*-actin, was also blotted, and a mean ratio was done to correct any differences in loading of the proteins. Our data showed no significant change in the densities of GLP-1 receptor bands between the normal and the treated groups (Figure 6).

## 4. Discussion

The GLP-1 receptor has been localized in many tissues, including the heart [4]. Recently, it was shown that this (GLP-1) receptor is absent in the mouse cardiac fibroblasts, but, abundant in the endocardium. Moreover, endothelium and coronary smooth muscle cells of the heart were also shown to bear GLP-1 receptors [18]. Upon binding to its receptor, GLP-1 produces numerous effects, but because of its very short physiologic half-life, its use as a therapeutic agent is not practical [19]. Incretin mimetics have been introduced in the market to be used as therapies for diabetes mellitus [20]. Our study aimed at assessing the modulation of GLP-1 receptor upon the usage of different treatment modalities including insulin, Exendin-4 (GLP-1 receptor agonist), and Aliskiren (potent renin inhibitor).

After one month of treatment, body weights of the rats in each group were recorded and our results showed a significant decrease in body weights of all diabetic rats compared to the normal. This can be explained by the process of muscle wasting that occurs in diabetic patients [21]. Interestingly, diabetic rats treated with Exendin-4 showed a significant increase in their body weight compared to the diabetic untreated (Figure 3). Moreover, Cardiac hypertrophy was assessed macroscopically (Figure 4); the ratio of heart weight to body weight revealed that there is a significant increase in most diabetic rats compared to that of the normal. This indicated the presence of cardiac hypertrophy in the diabetic rats. However, heart weight over body weight ratio of diabetic rats treated with Aliskiren showed no significance when compared to the ratio of normal. One can conclude that the beneficial effect of Aliskiren as a cardioprotective agent is manifested [13].

Blood glucose levels were extremely increased in all diabetic groups treated or untreated (Figure 5). The intravenous injection of streptozotocin (STZ)—a glucosamine-nitrosourea antibiotic with structural similarity to glucose that is readily taken up by the pancreatic *β* cells—causes *β* cells toxicity and ultimately necrosis, leading to deficiency in insulin secretion. The use of Streptozotocin to induce type 1 diabetes in rats is advantageous, because it is easy, fast, and the effect of diabetes on the heart can be evaluated [22]. Our aim was to assess the long-term effects of treatments on blood glucose level in type 1 diabetic rats. It is not surprising that after one month of insulin treatment blood glucose levels of the diabetic rats were not lowered/corrected, since insulin directly treats diabetes on a daily basis—as a short-term and not a long-term treatment. Insulin glargine may be expected to lower the blood glucose levels since it is a long-acting insulin; however, the duration of its action does not reach 24 hours with some recipients, which may be reflected by hyperglycemia [23]. Although not significant, Exendin-4 treatment showed slight improvement in the blood glucose levels. On the other hand, Aliskiren treatment significantly decreased blood glucose levels compared to the untreated. In fact, administration of Aliskiren alone normalized blood glucose levels of 12.5% of the treated rats. Recent studies have shown that Aliskiren improves insulin sensitivity in type 2 diabetic mice [24]; however, there are no reports on Aliskiren's effect on blood glucose in type 1 diabetes mellitus. The different combinations of Aliskiren with insulin or Exendin-4, however, exhibited no beneficial effects on the blood glucose levels compared to the normal.

GLP-1 levels in the plasma of normal rats showed a range of 7–9 pM with no significant variation throughout a month. Upon the induction of diabetes mellitus, however, GLP-1 levels increased with a maximum peak of 28 pM at day 28, compared to that of the normal. GLP-1 levels in diabetic rats treated with Insulin (DI), Exendin-4 (DE), or Aliskiren (DA) were normalized. The combination of Aliskiren with Insulin (DIA) was shown to be better than the combination of Exendin-4 and Aliskiren (DEA), because it corrected and nearly normalized GLP-1 levels in the diabetic rats. It is possible that the DPP-IV enzyme is upregulated in the diabetic state, thereby, inactivating GLP-1 molecules and reducing their affinity to the receptors. Hence, GLP-1 secretion is enhanced in the diabetic state to overcome this inactivation. Studies have reported that circulating DPPIV enzyme activity and mRNA are both enhanced in STZ-treated rats [25].

Rat heart perfusion technique showed an extremely significant decrease of GLP-1 affinity to its receptor in the diabetic state (D) compared to the normal (N) (1.14 ± 0.065 min versus 0.37 ± 0.007 min) at the level of the coronary endothelium. In diabetic rats treated with insulin (DI), GLP-1 affinity to its receptor increased beyond the normal (1.14 ± 0.065 min versus 2.17 ± 0.23 min), suggesting a direct effect of insulin on the GLP-1 receptor. Treatment with Exendin-4 (DE) (0.45 ± 0.01 min) slightly enhanced, while Aliskiren (DA) (0.38 ± 0.007) showed no significant change in its affinity compared to the diabetic (D) (0.37 ± 0.007 min). Interestingly, however, their combined therapy (DEA) (1.35 ± 0.0913 min) normalized the affinity constant. The cotreatment with Insulin and Aliskiren (DIA) showed some enhancement (0.657 ± 0.021 min) but could not be compared to the effect executed by (DEA) (1.35 ± 0.0913 min) (Table 1).

In parallel, it is interesting to note that the densities of GLP-1 receptors in the heart obtained by the Western blotting showed no difference between the normal or any of the diabetic-treated groups (Figure 6), while heart perfusion results showed differences in GLP-1 binding affinity between CM and CE in both the normal and the diabetic rats (Tables 1 and 2). At the level of the cardiomyocyte, our results showed a significant increase in the affinity of GLP-1 to its receptor in the diabetic compared to the normal and compared to the CHAPS-untreated diabetic. It is possible that GLP-1 receptors located on endothelial cells are different from those present on the cardiomyocytes, suggesting the existence of multiple subtypes of GLP-1 receptor, thereby explaining the difference in affinity constants in CHAPS-treated and -untreated diabetic rats. Another possible explanation could be that GLP-1 receptor expression is higher in the coronary endothelium than in cardiomyocytes. Insulin and Exendin-4 treatments showed slight improvement in the affinity (0.43 ± 0.009 min and 0.41 ± 0.0084 min, resp.), but Aliskiren and its combination treatments (DEA) and (DIA) did not lower the affinity (Table 2). 

These two findings could hint the possibility of different GLP-1 receptors existing in the coronary endothelium and cardiomyocytes. In fact, there have been discussions about the existence of a putative second GLP-1 receptor during a European GLP-1 Club Meeting in Marseille, where scientists have concluded that “although no molecular evidence has yet been presented, there are circumstantial data to suggest that such receptors do exist” [26]. 

According to Nystrom et al. [27], GLP-1 receptors are located on the human coronary endothelial cells. Binding of GLP-1 to those receptors was reported to induce vasodilation, probably mediated through Nitric Oxide production. Furthermore, this effect was eliminated upon the removal of the endothelial lining [26]. Our results showed a significant reduction of GLP-1 affinity to its receptor in the diabetic rat coronary endothelium compared to the normal. It is also probable that in the diabetic state, GLP-1 receptors on the coronary endothelial cells are modified in such a way that the binding affinity of GLP-1 to them is reduced, hence, resulting in reduced NO production and subsequent vasoconstriction. Moreover, this reduction in affinity was corrected beyond the normal by the treatment with insulin, suggesting a direct role of insulin on GLP-1 receptor modulation. The affinity constant in the diabetic rats was improved with Exendin-4 treatment, however Aliskiren did not ameliorate it. Interestingly, the combination of the two treatment modalities normalized the affinity suggesting synergy between the Exendin-4 and Aliskiren treatments. Recently, Dong et al. reported that, upon the administration of Aliskiren, the potent renin inhibitor, nitric oxide synthase production by endothelial cells was significantly restored [28]. In addition, GLP-1 infusion has been reported to induce vasodilation [26]. Combining Aliskiren and Exendin-4 probably causes an interaction, resulting in an exaggerated effect on the binding affinity of GLP-1 to its receptor on the coronary endothelium. 

## 5. Conclusions

Although many questions remain unanswered regarding the GLP-1 analogue, Exendin-4 and the renin-inhibitor, Aliskiren, it is evident that treatment with both Exendin-4, and Aliskiren greatly improves the GLP-1 binding affinity at the coronary endothelium level of the type 1 diabetic rat. It is therefore crucial to further investigate and to try and find a common ground between GLP-1 signaling pathway and the renin that could indicate a crosstalk between the two. Moreover, future investigations are crucial to unmask the possible existence of another GLP-1 receptor, hence explaining the difference in their affinities at the CE and CM levels. In conclusion, clinical studies on Aliskiren and/or its combination with Exendin-4 in type 1 diabetes mellitus could verify their long-term beneficiary effects on blood glucose levels and cardiomyopathy and further shed light on their ability to prevent the progression of diabetic complications especially in the heart.

## Figures and Tables

**Table 1:** The calculated dissociation constants (*K*_*d*_) and binding affinity constants (*τ*) of glucagon-like peptide-1 (GLP-1) with its receptor at the coronary endothelium (N = 8).

Rat Group	*K*_*d*_ (moles × 10^−15^)	*τ* (min)
Normal (N)	0.069 ± 0.005^a^	1.14 ± 0.065^a′^
Diabetic (D)	0.700 ± 0.029^b^	0.37 ± 0.007^b′^
Diabetic + Insulin (DI)	0.110 ± 0.025^c^	2.17 ± 0.23^c′^
Diabetic + Exendin-4 (DE)	0.580 ± 0.029^d^	0.45 ± 0.01^d′^
Diabetic +Aliskiren (DA)	0.857 ± 0.043^e^	0.38 ± 0.007^e′^
Diabetic + Exendin-4 + Aliskiren (DEA)	0.230 ± 0.039^f^	1.35 ± 0.0913^f′^
Diabetic + Insulin + Aliskiren (DIA)	0.650 ± 0.068^g^	0.657 ± 0.021^g′^

Coronary endothelium: dissociation constants significant at *P* < .05 for (a, f), (b, d), (b, e), (c, f) and *P* < .001 for (a, b), (a, d), (a, e),(a, g), (b, c), (b, f), (c, d), (c, e), (c, g), (d, e), (d, f), (e, f), (f, g). The other comparative values are not significant (*P* > .05). Binding affinities significant at *P* < .05 for (c′, f′) and *P* < .001 for (a′, b′), (a′, c′), (a′, d′), (a′, e′), (a′, g′), (b′, c′), (b′, d′), (b′, f′), (b′, g′), (c′, d′), (c′, e′), (c′, g′), (d′, e′), (d′, f′), (d′, g′), (e′, f′), (e′, g′), (f′, g′). The other comparative values are not significant (*P* > .05).

**Table 2:** The calculated dissociation constants (*K*_*d*_) and binding affinity constants (*τ*) of glucagon-like peptide-1 (GLP-1) with its receptor at the cardiomyocytes (N = 8).

Rat group	*K*_*d*_ (moles × 10^−15^)	*τ* (min)
Normal (N)	0.64 ± 0.021^a^	0.34 ± 0.005^a′^
Diabetic (D)	0.33 ± 0.018^b^	0.6 ± 0.018^b′^
Diabetic + Insulin (DI)	0.47 ± 0.019^c^	0.43 ± 0.009^c′^
Diabetic + Exendin-4 (DE)	0.59 ± 0.025^d^	0.41 ± 0.0084^d′^
Diabetic + Aliskiren (DA)	0.12 ± 0.014^e^	1.37 ± 0.0938^e′^
Diabetic + Exendin-4 + Aliskiren (DEA)	0.077 ± 0.01^f^	1.63 ± 0.13^f′^
Diabetic + Insulin + Aliskiren (DIA)	0.19 ± 0.027^g^	1.33 ± 0.08^g′^

Cardiomyocytes: dissociation constants significant at *P* < .05 for (b, c), (b, g), (c, d), (e, f), (f, g) and *P* < .001 for (a, b), (a, c), (a, e), (a, f), (a, g), (b, d), (b, e), (b, f), (c, e), (c, f), (c, g), (d, e), (d, f), (d, g), (f, g). The other comparative values are not significant (*P* > .05). Binding affinities significant at *P* < .001 for (a′, b′), (a′, c′), (a′, d′), (a′, e′), (a′, f′), (a′, g′), (b′, c′), (b′, d′), (b′, e′), (b′, f′), (b′, g′), (c′, e′), (c′, f′), (c′, g′), (d′, e′), (d′, f′), (d′, g′). The other comparative values are not significant (*P* > .05).
